# Improving self-efficacy through nurse navigation in patients during treatment for colorectal cancer: a randomized controlled trial

**DOI:** 10.1007/s00520-026-11035-9

**Published:** 2026-08-06

**Authors:** Marianne Kirstine Thygesen, Sonja Wehberg, Karin B. Dieperink, Jens Søndergaard, Sharaf K. Perdawood, Niels Qvist

**Affiliations:** 1 Reserch Unit for Gynaecology and Obstetrics, Odense University Hospital, University of Southern Denmark, Odense, Denmark; 2 Research Unit of General Practice, Department of Public Health, Faculty of Health Sciences, University of Southern Denmark, Odense, Denmark; 3 Research Unit of Oncology, Family Focused Healthcare Research Centre (FaCe), Odense University Hospital, University of Southern Denmark, Odense, Denmark; 4 Department of Surgery, Slagelse Hospital, Slagelse, Denmark; 5 Research Unit for Surgery, Odense University Hospital, University of Southern Denmark, Odense, Denmark

**Keywords:** Nurse navigation, Colorectal cancer, Cancer Behavior Inventory questionnaire, Self-efficacy, Randomized controlled trial

## Abstract

**Purpose:**

We hypothesized that a nursing navigator intervention with needs assessment, distress evaluation, and emotional support from diagnosis to follow-up would improve self-efficacy compared with standard care in colorectal cancer.

**Methods:**

Patients with a physician-verified suspicion of colorectal cancer following colonoscopy were assessed for eligibility. Those who fulfilled inclusion criteria and consented were randomized to either intervention (nurse navigation) or standard care during the following cancer treatment trajectory. Five special educated nurse navigators delivered the intervention. Self-efficacy was assessed using the short form of the Cancer Behavior Inventory questionnaire (CBI-B) at baseline before randomization and at treatment completion or within 1 year at the latest. The group difference between CBI-B changes from baseline to follow-up was assessed in a population-averaged linear regression model employing generalized estimating equations.

**Results:**

Of 271 included participants, 222 were completed cases (115 in standard care, 107 in intervention). Based on 4888 observations, self-efficacy under standard care increased by a 2.06 score (CI −0.84; 4.97) and by 6.98 (CI 3.99; 9.97) under intervention. Estimated effect size was 4.92 (CI 0.75; 9.08), which was statistically significant (*p* = 0.021).

**Conclusions:**

Tailored nurse navigation across the course of colorectal cancer treatment significantly increased patients’ self-efficacy.

**Supplementary Information:**

The online version contains supplementary material available at https://doi.org/10.1007/s00520-026-11035-9.

## Background

Cancer diagnoses profoundly disrupt patients’ lives, triggering psychological stress that can persist for years. A register-based study from Sweden, whose healthcare system is similar to the Danish system, found that the risk of mental disorders begins to rise approximately 10 months before a cancer diagnosis, peaks during the first week after confirmation, and, although it decreases thereafter, remains elevated up to 10 years post-diagnosis [1].

The diagnostic phase marks the start of the cancer journey, during which patients rely on personal resilience and support networks. Self-efficacy is central in managing distress in cancer care. According to Bandura, self-efficacy reflects beliefs that underpin motivation, well-being, and personal fulfillment in specific contexts, such as navigating through the trajectory of a cancer diagnosis and treatment [2]. Patients with higher self-efficacy experience less pain and fatigue [3, 4], and with greater overall well-being and quality of life [5, 6]. Measures to enhance self-efficacy may include psychosocial care, support from family, friends, patient organizations, psychologists, and healthcare professionals [7, 8].

Self-efficacy can be assessed using validated instruments, and for coping with cancer, the brief version of the Cancer Behavior Inventory (CBI-B) has been validated and widely used [9]. The Canadian nurse navigation can be used to identify patients who may benefit from interventions or require referral to psychosocial resources [10]. An important factor to develop a high level of self-efficacy during treatment are healthcare professionals who are devoted to maintain continuous contact with patients [11]. Special educated nurse navigators might be the most appropriate through their special knowledge and ability to interact within the healthcare system. By a maintained direct contact with the patient, they can undertake tasks that address patients’ emotional support and education [12, 13]. The concept of nurse navigation has been shown to enhance patient adherence to both examinations and treatment, reduce the length of hospital stay, and alleviate patient anxiety in some cohort studies [11, 14, 15], but not in others [16, 17]. Overall, precise guidance on psychosocial care to enhance self-efficacy in patients with cancer is lacking.

We aimed to test whether adding a specially designed nurse navigation to standard care, from the diagnosis of colorectal cancer to the end of treatment or 1 year after inclusion, would be superior to standard care in terms of changes in patient-reported self-efficacy.

## Methods

### Trial design, participants, and randomization

Patients with a suspected colorectal cancer after colonoscopy were recruited from the endoscopy unit at the two hospitals. The study was conducted as a non-blinded randomized clinical trial comparing two arms: standard care versus an intervention targeting integrated psychosocial care. The person (SW) who performed data analysis was blinded to the group allocation. A detailed description of the protocol has been published previously [18].

Patients with colorectal cancer were selected, as this is a common cancer type with a relatively favorable prognosis. Eligible participants were 18 years or older with a physician-verified suspicion of colorectal cancer following colonoscopy. Exclusion criteria included being in the diagnostic phase of, or diagnosed with, dementia, severe psychiatric illness, intellectual disability, an American Society of Anesthesiologists (ASA) score of IV or greater, and inability to speak or understand Danish. After written informed consent was obtained, patients were randomized to either intervention or control group (standard care). Randomization was done centrally (web-based hosted by the Open Patient Data Explorative Network (OPEN) in two equal groups [ratio 1:1], with stratification on gender and age (18–69 versus 70 +). This strategy was chosen to provide an even distribution of variables across the two study groups to minimize accidental confounding as self-efficacy may relate to age and gender. Participants were asked to fill out questionnaires at two time points: at baseline/before randomization (T1) and at treatment completion or at the latest 1 year after inclusion (T2) in those who had not completed treatment within 1 year due to a predefined follow-up on a maximum of 1 year.

### Setting

We recruited participants from two Danish hospitals: Odense University Hospital and the University Hospital in Slagelse, during the period from February 16, 2016, to October 23, 2017, with follow-up until November 1, 2018. Both hospitals serve as primary referral center for colonoscopy covering 660,000 inhabitants from cities, rural areas, and remote islands. Referral to colonoscopy was either symptoms suspicious for colorectal cancer or individuals with a positive fecal occult blood test (FOBT) through participation in the Danish national screening program for colorectal cancer, which is offered to all Danish individuals aged 50 to 74 years. All patients with a confirmed histological adenocarcinoma at colonoscopy enter a standardized diagnostic program followed by regular multidisciplinary team conferences with a plan for treatment.

### Standard care

Standard care included in-house booking staff who were available by phone during office hours, 5 days a week, to answer questions related to the specific course of treatment and appointments. If patients received treatment across more departments, the assigned in-house booker would change accordingly. Booking staff could also contact patients directly to inform them of short-notice appointments. Additionally, standard care involved a post-treatment follow-up call from a nurse that included a structured needs assessment focused on the patient’s health-related concerns and continued care within the hospital system.

### Intervention care

**Fig. 1:**
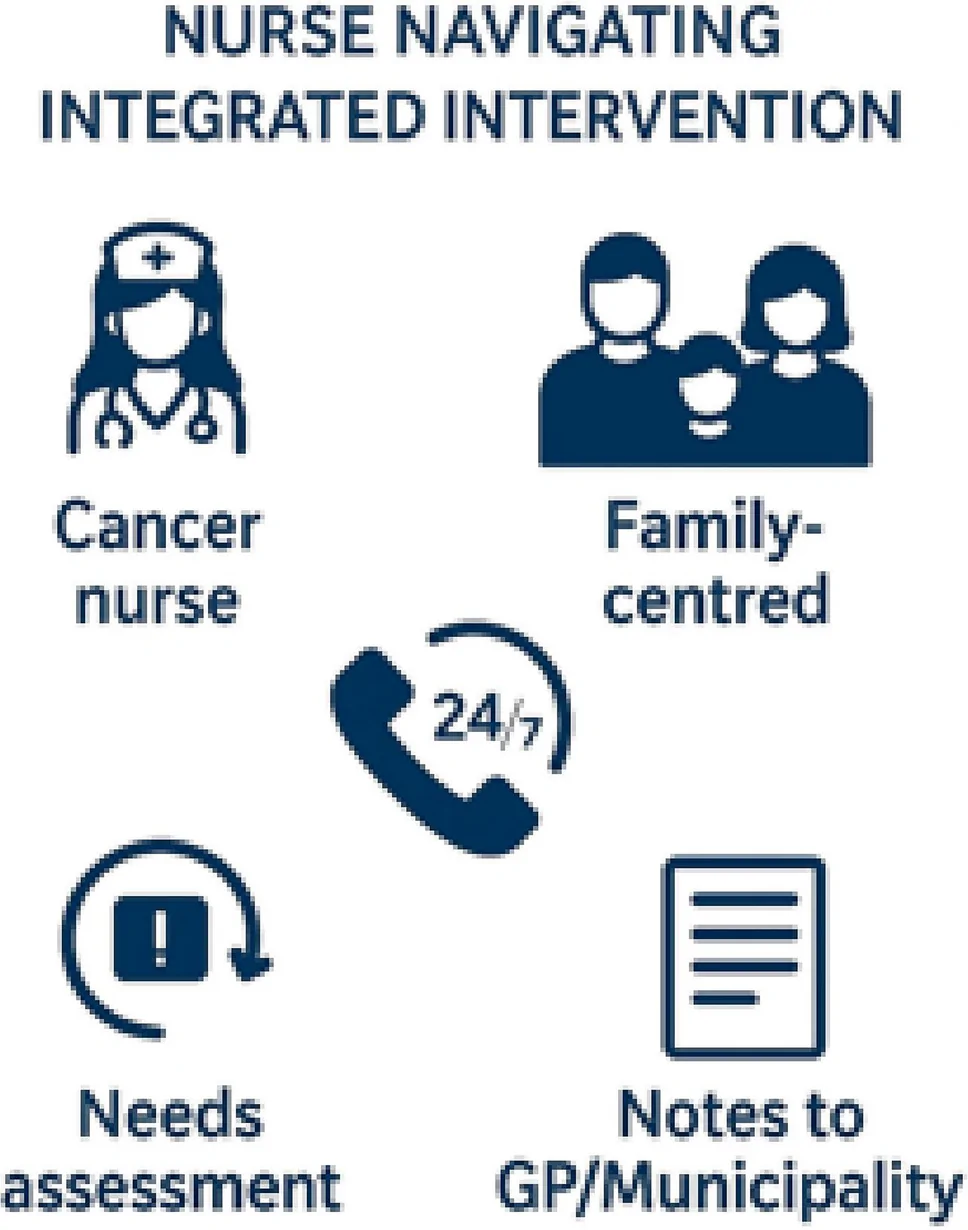
Core elements in the nurse navigator intervention

The intervention involved linking the patient to one of five proactive, patient-specific nurse navigators who provided continuity and remote support throughout the entire trajectory in addition to the standard care. Patients could contact the nurse navigator at any time by leaving a message on the answering machine, and she would return the call within 4 h during office hours (Fig. 1). This accessibility was intended to establish the nurse navigator as a significant and trusted figure, encouraging patients to actively engage in coping with their situation. In case, the patient did not contact the nurse navigator, she was proactive and contacted the patient at four time points: (1) within 72 h after inclusion, (2) before cancer treatment start, (3) 7 to 10 days after initiation of cancer treatment, and (4) 1 week later, or when an additional treatment eventually was initiated. Intervention continued until completion of the questionnaire after end of treatment or at 1 year as the latest (T2).

The nurse navigators were recruited among experienced nurses from the surgical or oncological ward and they all went through a standardized 1-week educational program within the concepts and topics including nurse navigation, self-efficacy, and family nursing. Nurse navigators were educated to listen attentively, to provide positive reinforcement for patients’ coping efforts, to encourage continued engagement, and to offer relevant psychosocial support. In the screening for distress and global unmet needs, the validated assessment tool from the Canadian nurse navigation and adjusted to the Danish setting following a back-and-forth translated approach was used [10]. It includes a distress score (VAS), a symptom list, and the Edmonton Symptom Assessment Scale (ESAS). Patients that scored VAS > 5/10 for distress or ESAS on > 7/10 were referred to the general practitioner as a safety procedure and not at part of the nurse navigation.

To maintain the intervention as intended, the first author (MKT) conducted regular audits of nurse navigators’ notes and voice-recorded communications with selected patients. This made it possible to discuss what was done, how it was done, and how it related to the overall aim of nurse navigation.

### Outcomes

The brief Cancer Behavior Inventory (CBI-B) including 12 items was used for measuring self-efficacy as our primary outcome [9]. All items are scored on a nine-point Likert scale from 1 “not at all confident” to 9 “very confident.” The scores from all items are summarized to a total range from 12 to 108. Single missing items were not imputed. Consequently, if a respondent left any item unanswered, the CBI-B score for that time point was handled as missing. The primary outcome was the difference between randomization groups in changes of CBI-B from baseline (T1) to end of follow-up (T2). Exploratory outcomes were the CBI-B score in subgroups with surgery alone, neoadjuvant or adjuvant chemotherapy, and healthcare professionals among relative.

### Analysis

#### Power calculation and statistical analysis

An increase change in self-efficacy (primary outcome) measured with the CBI-B questionnaire on 8 points was considered clinically relevant. With an assumption of a CBI-B total score on 88 at baseline and with a standard deviation of 20, a significant level on 0.05, and a power on 80%, 100 patients must be included in each arm. With an anticipated dropout on approximately one third, a total of 280 patients was planned to be included.

The number of non-missing and missing observations by time point, as well as the number of complete cases, defined as participants with observations at both time points was calculated. To illustrate the intervention effect on the primary outcome, CBI-B, we calculated individual differences in total scores between T2 (follow-up) and T1 (baseline) for completed cases. A histogram with overlaid estimated normal densities was created to illustrate the outcome effect.

We analyzed the efficacy of the intervention using a population-averaged linear regression model based on generalized estimating equations (GEE), where the effect was estimated as a group-time interaction term. This approach was chosen instead of the analysis of covariance (ANCOVA) specified in the protocol, to account for missing observations and include participants with either missing baseline or follow-up data. Under the assumptions of missing completely at random (MCAR) for observations missing at baseline and MCAR conditioning on baseline and treatment (group status) for observations missing at follow-up, this approach yields sensible estimates. We present the frequency of missing values separately for both time points and include a multivariable logistic regression model for the outcome “completion” at T2.

Additionally, we present results from ANCOVA and a two-sample *t*-test on the individual differences, both conducted on complete cases. Also, for complete cases only, group differences were described by Cohen’s d.

As post hoc analysis, we employed the same analysis strategy in the following subgroups based on treatment characteristics and caregiver involvement: (1) patients treated with surgery alone, (2) patients who received neoadjuvant or adjuvant oncological treatment, and (3) patients with healthcare professionals among relatives.

### Ethical considerations

This study follows the guidelines of Helsinki and is registered with number 19/26956 at the Danish Data Protection Agency, ID NCT03281447 at Clinicaltrial.gov (registration date 11.09.2017), and was approved by the Regional Committees on Health Research Ethics for Southern Denmark (S-20150120). All patients signed informed consent. As an additional patient safety, for any unexplained gaps in the trajectory (diagnostic or treatment), an in-house booker was informed to take proper action.

## Results

### Participant flow and numbers analyzed

**Fig. 2:**
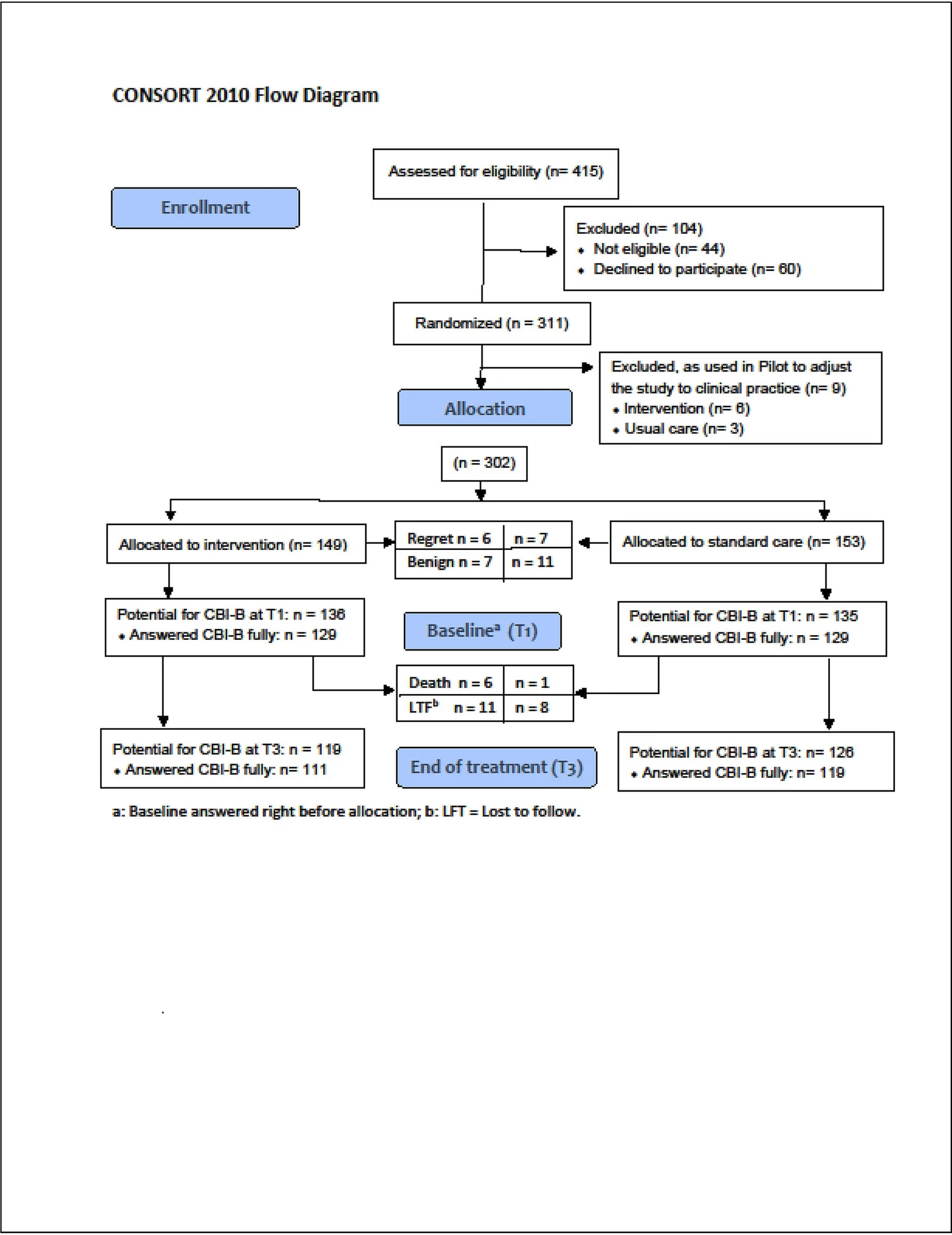
Flowchart of included patients

Out of 415 eligible patients, 44 did not fulfil the inclusion criteria, and 60 participants declined due to lack of mental resources to participate. Thus, a total of 311 fulfilled the inclusion criteria (Fig. 1). The first nine patients included were used to test study design and to allow for adjustment of the intervention in relation to clinical practice. These patients were excluded from the final analysis. Thus, a total of 302 patients was subsequently randomized (intervention group: *n* = 149; standard care group: *n* = 153). Thirteen participants (intervention: *n* = 6; standard care: *n* = 7) withdrew their consent after randomization without providing a reason. Eighteen patients turned out to have a benign disease at final histological examination and were excluded from the analysis. During the follow-up period, seven died, and 19 patients were lost in follow-up for various reasons (Fig. 2).

Thus, a total of 271 patients were included in the study. The number of patients with missing CBI-B at baseline (T1) and at follow-up (T2) are shown in Table 1, and 222 patients (intervention *n* = 107 and standard care *n* = 115) adhered to the protocol complete the study. For the primary analysis, 266 patients contributed a total of 488 observations of CBI-B, either at baseline and/or at T2 (Supplementary Table S2). An overview of non-participation at baseline (*n* = 13) and follow-up (*n* = 36) in relation to patient factors is presented in supplementary tables S3 and S4. No significant differences were found.

**Table 1:** Number of patients (*N*) included and number of patients with missing Cancer Behavior Inventory (CBI-B) observations at baseline (T1) and follow-up (at end of treatment or at the latest one year after inclusion (T2) in the intervention group with nurse navigation and control group with standard care. Corresponding information for all post-hoc subgroups is available in supplementary table S1

	**At baseline (T1)**			**At follow-up (T2)**			**Completed cases**
	*N*	Missing CBI-B	Included in analysis	N	Missing CBI-B	Included in analysis	N
**Overall**	271	13	258	245	15	230	222
Control	135	6	129	126	7	119	115
Intervention	136	7	129	119	8	111	107
**Restricted to patients treated with surgery, only**							
Control	70	1	69	67	0	67	67
Intervention	73	3	70	69	3	66	63

### Baseline data

Sociodemographic data of the included patients revealed a balanced distribution between groups and with no significant differences (Table 2). Overall, compared to the standard care group, the intervention group had less patients with chronic diseases, less with colon cancer, and more patients were cancer free at the end of treatment.

**Table 2:** Socio-demographic characteristics of all 271 participants included and by randomization group. Figures are absolute numbers with percentages in brackets

	**All included** ***N* = 271**	**Control** ***N* = 135**	**Intervention** ***N* = 136**
**Gender** (Male)	145 (53.5)	74 (54.8)	71 (52.2)
**Years of age**			
< 55	26 (9.6)	12 (8.9)	14 (10.3)
55–69	118 (43.5)	58 (43.0)	60 (44.1)
≥ 70	127 (46.9)	65 (48.1)	62 (45.6)
**Highest education level (**years)			
≤ 10	54 (19.9)	31 (23.0)	23 (16.9)
11–14	57 (21.0)	27 (20.0)	30 (22.1)
15–16	57 (21.0)	26 (19.3)	31 (22.8)
> 16	20 (7.4)	9 (6.7)	11 (8.1)
Vocational	83 (30.6)	42 (31.1)	41 (30.1)
Living alone	78 (28.8)	39 (28.9)	39 (28.7)
Having children	77 (28.4)	40 (29.6)	37 (27.2)
Comorbidity: At least one chronic disease	96 (35.4)	53 (39.3)	43 (31.6)
**Cancer location**			
Colon	190 (70.1)	98 (72.6)	92 (67.6)
Rectum	81 (29.9)	37 (27.4)	44 (32.4)
Cancer free at follow-up	251 (92.6)	122 (90.4)	129 (94.9)

### Primary outcome (CBI-B)

Patients in standard care group (controls) increased their self-efficacy score non-significantly from baseline to follow-up on average by 2.06 (95% confidence interval (CI) −0.84; 4.97), while patients in the intervention group showed a statistically significant mean increase of 6.98 (95% CI 3.99; 9.97). The intervention increased patients’ self-efficacy to a higher level, with an estimated group difference in CBI-B change of 4.92 (*p* = 0.021) (95% CI 0.75; 9.08) as shown in Table 3. Secondary analyses, i.e., alternative statistical analyses, resulted in similar *p*-values. The total time spent on navigation was less than 30 min on average for each patient in the intervention group.

**Table 3:** Primary outcome CBI-B: Efficacy of the intervention based on a GEE population-averaged linear regression, where the effectiveness is estimated as a group-time interaction term. In addition, we present mean scores and standard deviation (SD) in each group at baseline (T1) and follow-up (T2)

	**Baseline (T1)**		**Follow-up (T2)**		**Estimated change within groups (95% CI)**^**a**^	**Group-time interaction (95% CI)**^**b**^	***p*-value**	***p*-value based on *t*-test**^**c**^	***p*-value based on ANCOVA**^**d**^
	Number of patients	CBI-B mean (SD)	Number of patients	CBI-B mean (SD)					
Control	129	80.2 (16.6)	119	82.3 (18.4)	2.06 (− 0.84;4.97)				
Intervention	129	79.0 (16.8)	111	86.1 (16.0)	6.98 (3.99; 9.97)	4.92 (0.75; 9.08)	0.0208	0.0300	0.0397

^a^For the control group, this within-group change is the estimated regression coefficient for time (follow-up versus baseline); for the intervention group, the within-group change is based on the estimated coefficient for time and the estimated group-time interaction

^b^The group effect is modelled as the difference between the within-group changes over time, that is, the coefficient of the group-time interaction term

^c^Secondary analysis: *p*-value based on a two-sample *t*-test on the individual changes, calculated on complete cases

^d^Secondary analysis: *p*-value corresponding to the estimated group coefficient in a linear regression model on CBI-B at T2 adjusted for baseline (ANCOVA), calculated on complete cases

**Fig. 3:**
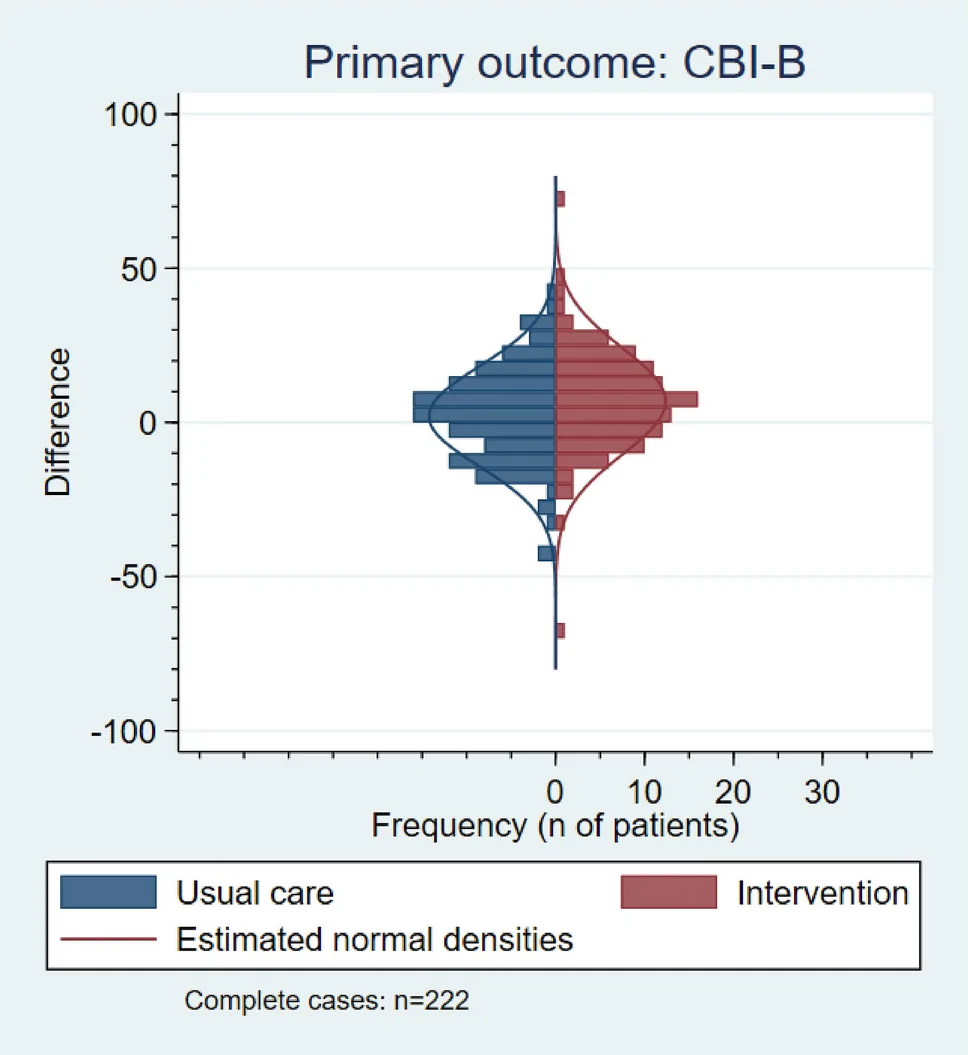
Illustration of the primary outcome: distribution of individual difference in CBI-B (end measurement minus baseline measurement) in complete cases by randomization group

In an additional analysis restricted to patients with both observations (complete cases) only, the effect size (Cohen’s d) was 0.29, with a corresponding *p*-value for the *t*-test of *p* = 0.030 (Table S5). An illustration of the estimated group difference, again restricted to complete cases, can be seen in Fig. 3.

An illustration of the group differences is shown in Fig. 3.

### Explorative post hoc subgroup analyses

More than half of the patients were treated with surgery only (Table 4), and those who received the intervention had a 5.10 points higher increase in self-efficacy compared to those with standard care (*p* = 0.080) (95% CI −0.61; 10.82). For those who underwent neoadjuvant or adjuvant oncological treatment, the difference in the increase in self-efficacy was 4.66 points (*p* = 0.136) (95% CI 1.37; 13.80). For participants with a healthcare professional among relatives, a statistically significant increase in self-efficacy was found in the intervention group, only with an increase of 7.59 point (*p* = 0.017) (95% CI 1.37; 13.80).

**Table 4:** Explorative post-hoc subgroup analysis of patients with surgery alone, adjuvant and neoadjuvant therapy and patients with healthcare professionals among relatives. Efficacy of the intervention is evaluated in separate GEE population-averaged linear regression models, where the effectiveness is estimated as a group-time interaction term. In addition, we present mean scores and standard deviation (SD) in each randomization group at baseline (T1) and follow-up (T2), for each subgroup

	**Baseline (T1)**		**Follow-up (T2)**		**Estimated change within groups (95% CI)**^**a**^	**Group-time interaction (95% CI)**^**b**^	***p*-value**	***p*-value (*t*-test**^**c**^**)**	**p-value (ANCOVA**^**d**^**)**
	**Number of patients**	**CBI-B mean (SD)**	**Number of patients**	**CBI-B mean (SD)**					
**Patients who underwent surgery, alone**									
Control	69	78.3 (15.6)	67	81.4 (18.8)	2.81 (− 1.20; 6.82)				
Intervention	70	77.4 (17.8)	66	85.5 (18.2)	7.91 (3.83; 11.98)	5.10 (− 0.61; 10.82)	0.0802	0.0904	0.1060
**Patients who underwent neoadjuvant or adjuvant therapy**									
Control	60	82.3 (17.6)	52	83.5 (18.0)	1.17 (− 3.07; 5.41)				
Intervention	59	81.0 (15.3)	45	87.0 (12.3)	5.83 (1.40; 10.26)	4.66 (− 1.47; 10.79)	0.1361	0.1818	0.2110
**Patients with a healthcare professional among relatives**									
Control	58	82.3 (15.6)	55	81.8 (19.5)	0.14 (− 4.00; 4.27)				
Intervention	49	81.0 (14.5)	42	89.4 (11.5)	7.73 (3.09; 12.37)	7.59 (1.37; 13.80)	0.0167	0.0593	0.0300

^a^For the control group, this within-group change is the estimated regression coefficient for time (follow-up versus baseline); for the intervention group, the within-group change is based on the estimated coefficient for time and the estimated group-time interaction

^b^The group effect is modelled as the difference between the within-group changes over time, that is, the coefficient of the group-time interaction term

^c^Secondary analysis: *p*-value based on a two-sample *t*-test on the individual changes, calculated on complete cases

^d^Secondary analysis: p-value corresponding to the estimated group coefficient in a linear regression model on CBI-B at T2 adjusted for baseline (ANCOVA), calculated on complete cases

A comparison between patients treated with a curative intention and those who received palliative treatment only was not possible due to a low number of patients on palliative treatment.

## Discussion

Our intervention involving nurse navigation significantly improved self-efficacy among patients undergoing treatment for colorectal cancer, given that self-efficacy is positively associated with well-being, quality of life, and optimism, and adversely associated with depression, sickness impact, pain, and fatigue [3, 4, 19, 20]. Although self-efficacy is recognized as a mediator for mental health in psychiatry [21], our study did not specifically examine this aspect, nor did it explore the potential relationship between self-efficacy and quality of life [22]. At follow-up, patients receiving the intervention reported a greater increase in CBI-B scores compared to those in standard care, with an effect size of 0.29. There is no universal accepted fixed threshold for the Cohen’s d that determines clinical relevance. It is context-dependent and usually requires a value between 0.2 and 0.8 depending upon the severity of the disease and the risk associated with its treatment [23]. Despite modest value in our study, this effect was considered clinically meaningful given the complexity of the cancer trajectory, the psychosocial challenges patients faced during treatment, and the relative low-cost intervention. Our findings align with previous studies in other cancer diagnoses that nurse navigation and individualized supportive care can strengthen patients’ coping abilities and self-management [24].

A similar increase in self-efficacy among those who underwent surgical treatment only and in those who had neoadjuvant or adjuvant oncological treatment was found. We believe that this may indicate that nurse navigation addressed more than just the information gaps during transitions, as reported by others [25–27].

Our intervention was grounded in additional healthcare contact, including needs assessments and continuity of care. While some elements of this were present in standard care, particularly for patients who received a follow-up call from a nurse, our findings suggest that nurse navigation offered more comprehensive support. The early establishment of a professional relationship during a period of heightened distress, proactive engagement before the first treatment, sustained continuity, and a focus on enhancing self-efficacy by addressing distressing issues may collectively explain the observed benefits.

Patients with a healthcare professional among their caregivers derived particularly strong gains from the intervention, suggesting that navigation may amplify the impact of existing supportive resources. Similarly, the positive trends observed for both surgically treated patients and those undergoing combined oncological treatment point to the adaptability of the intervention across different treatment trajectories. While not all subgroup findings reached statistical significance, the consistent direction of effects favors the intervention and warrants further exploration in larger samples.

A recent review found that nurse navigator programs have shown to improve cancer patients’ experiences and the efficiency of cancer care delivery [28]. Integration of nurse navigation into the healthcare team necessitates longitudinal financial and professional support. However, resource constraints in hospitals may have led to a prioritization of health equity over equal care, as seen in public health [29]. It may well have been the case that healthcare professionals provided more support to patients without a healthcare professional in their personal network, inadvertently leaving others less health literate. Future research should explore how the principle of health equity affects both patients and caregivers.

## Strengths and limitations

We assessed outcomes at comparable milestones in the cancer trajectory: at the point of physician-verified suspicion (baseline) and at the end of treatment or 1 year post-inclusion for those still undergoing treatment. Given the variability in cancer courses, we believe this event-based timing enhances internal validity compared to studies using fixed intervals (e.g., 3 or 6 months post-baseline) [30, 31].

There could be a potential source of bias if nurse navigators influenced physicians to allocate more attention to their patients in the intervention group, thereby diverting resources from the control group. To mitigate this, nurse navigators were restricted to participate in MDT conferences. The difference in length of follow-up might be a possible confounder, but the number of patients who completed the questionnaire at 1 year or treatment completion was comparable between the two groups.

Another limitation relates to the nature of self-efficacy measurement. There is a risk that patients with declining self-efficacy may drop out of the study to focus on their personal well-being, potentially skewing results. However, evidence suggests that patients with low self-efficacy benefit more from psychological interventions than those with high self-efficacy [32], and other studies have documented declines in self-efficacy during cancer trajectories [33, 34]. The intervention involved increased patient contact and support compared to standard care. This raises the possibility that the observed effects may reflect attention or contact bias rather than the specific components of nurse navigation.

We did not track potential harm, which is a limitation. The intervention may have inadvertently heightened patients’ focus on their illness, contributing to attrition. Our study included adult patients with a cancer type that is curable in early stages and manageable in advanced stages.

Data collection was performed almost 10 years ago which might be a limitation as the field of nurse navigation has evolved rapidly during the last decade as well as treatment and patient trajectory may have changed.

## Conclusions

In patients with colorectal cancer, a dedicated nurse navigation significantly improved self-efficacy compared to standard care. The average time spent on each patient was under 30 min, resulting in a low-cost intervention. Further studies should examine how to implement nurse navigation in daily clinic and whether this may improve quality of life in survivors and or improvement in the long-term prognosis.

### Participation

MKT, KBD, and JS wrote the protocol. The nurse navigation concept was developed in Ontario, Canada. MKT specified the psychological part of the intervention, and MKT and KBD partly educated the nurse navigators. MKT followed the intervention and held bimonthly feedback sessions with nurse navigators to ensure the intervention. MKT, KBD, SKP, and NQ ensured randomization and data gathering. SW did the statistical analysis, and all authors were involved in interpretation of results and manuscript editing.

## Supplementary Information

Below is the link to the electronic supplementary material.

ESM1 (DOCX 29.2 KB)

## Data Availability

The data are available upon request to the corresponding author.
