# Supplementary material for: Improving self-efficacy through nurse navigation in patients during treatment for colorectal cancer: a randomized controlled trial
Source: Support Care Cancer. 2026 Aug 6;34(9):830. doi: 10.1007/s00520-026-11035-9 (PMC13442497; doi:10.1007/s00520-026-11035-9)
Supplement: Supplementary file 1 [file 520_2026_11035_MOESM1_ESM.docx]

# Supplementary Material

**Table S1. Post-hoc subgroups. Overview of number of observations for CBI-B by time and intervention group (Baseline and follow-up/end of treatment). Potential participants at T2 are all study participants who did not end their study participation between T1 and T2 (see Flowchart, Figure 2).**

| **Groups** | **At Baseline (T1)** | | | **At End of treatment (T2)** | | | **CC*** |
| --- | --- | --- | --- | --- | --- | --- | --- |
|  | N | Missing CBI-B | Analysis | Potential participation | Missing survey  or missing CBI-B | Analysis |  |
| **Patients treated with solely surgery** |  |  |  |  |  |  |  |
| Usual care | 70 | 1 | 69 | 67 | 0 | 67 | 67 |
| Intervention | 73 | 3 | 70 | 69 | 3 | 66 | 63 |
| **Patients treated without surgery** |  |  |  |  |  |  |  |
| Usual care | 65 | 5 | 60 | 59 | 7 | 52 | 48 |
| Intervention | 63 | 4 | 59 | 50 | 5 | 45 | 44 |
| **Patients with healthcare professional among relatives (yes)** |  |  |  |  |  |  |  |
| Usual care | 61 | 3 | 58 | 59 | 4 | 55 | 52 |
| Intervention | 51 | 2 | 49 | 45 | 3 | 42 | 41 |

* CC: Complete cases

**Table S2. Overview of number of contributed observations per participant by intervention group.**

|  | **N** | **Analysis at baseline (T1)** | **CBI-B measurement available at** |  |  |  | **Included observations in main analysis** |
| --- | --- | --- | --- | --- | --- | --- | --- |
|  |  |  | Only baseline | Only follow-up | Both times | Never |  |
| **Overall** | **271** | **258** | **36** | **8** | **222** | **5** | **444+36+8 = 488** |
| Usual care | 135 | 129 | 14 | 4 | 115 | 2 | 248 |
| Intervention | 136 | 129 | 22 | 4 | 107 | 3 | 240 |

**Table S3. Non-participation at baseline (N=13 patients: N=6 usual care and N=7 intervention). Non-participation at baseline covers included patients (N=271) who never contributed a valid CBI-B measurement (N=5) or only contributed one at follow-up (N=8).**

To prevent participant identifiability in connection to patient characteristics, cells containing less than three patients are only indicated as “<3”.

|  | **All included** | **Baseline available** | **Non-participation at baseline** |
| --- | --- | --- | --- |
|  | **N** | **N (row %)** | **N (row %)** |
| **All** | **271** | 258 (95.2) | 13 (4.8) |
| **Randomization group** |  |  |  |
| Usual care | 135 | 129 (95.6) | 6 (4.4) |
| Intervention | 136 | 129 (94.9) | 7 (5.1) |
| **Gender** |  |  |  |
| Men | 145 | 141 (97.2) | 4 (2.8) |
| Women | 126 | 117 (92.9) | 9 (7.1) |
| **Years of age** |  |  |  |
| 33-54 | 26 | 26 (100.0) |  |
| 55-69 | 118 | 113 (95.8) | 5 (4.2) |
| 70-91 | 127 | 119 (93.7) | 8 (6.3) |
| **Highest education level**  **(years)** |  |  |  |
| ≤ 10 | 54 | 51 (94.4) | 3 (5.6) |
| 11-14 | 57 | 53 (93.0) | 4 (7.0) |
| 15-16 | 57 |  | <3 |
| >16 | 20 |  | <3 |
| Vocational | 83 | 79 (95.2) | 4 (4.8) |
| **Living alone (yes)** | 78 | 69 (88.5) | 9 (11.5) |
| No | 193 | 189 (97.9) | 4 (2.1) |
| **Having children (yes)** | 77 | 74 (96.1) | 3 (3.9) |
| No | 194 | 184 (94.8) | 10 (5.2) |
| **Comorbidity: At least one**  **chronic condition** | 96 | 91 (94.8) | 5 (5.2) |
| Not | 175 | 167 (95.4) | 8 (4.6) |
| **Cancer location** |  |  |  |
| Colon | 190 | 180 (94.7) | 10 (5.3) |
| Rectum | 81 | 78 (96.3) | 3 (3.7) |
| **Cancer free at follow-up** | 251 (92.6) | 239 (95.2) | 12 (4.8) |
| No | 20 | 19 (95.0) | 1 (5.0) |

**Table S4. Completers (N=222) versus non-completers (N=36) of participants included at baseline (N=258). Presented are N and row percentages per group. In addition, we include odds ratios (OR) with corresponding 95% confidence intervals (95% CI) based on a multivariable logistic regression model with “completion” as outcome.**

To prevent participant identifiability in connection with patient characteristics, cells containing less than three patients are only indicated as “<3”.

|  | **Included**  **at baseline** | **Complete cases** | **Non-completers** |  |
| --- | --- | --- | --- | --- |
|  | **N** | **N (row %)** | **N (row %)** | **OR (95% CI)** |
| **All** | 258 (100.0) | 222 (86.0) | 36 (14.0) |  |
| **Randomization group** |  |  |  |  |
| Usual care | 129 | 115 (89.1) | 14 (10.9) | Ref |
| Intervention | 129 | 107 (82.9) | 22 (17.1) | 0.45 (0.21-1.00) |
| **Gender** |  |  |  |  |
| Men | 141 | 120 (85.1) | 21 (14.9) | Ref |
| Women | 117 | 102 (87.2) | 15 (12.8) | 1.54 (0.67-3.58) |
| **Years of age** |  |  |  |  |
| 33-54 | 26 | 23 (88.5) | 3 (11.5) | Ref |
| 55-69 | 113 | 104 (92.0) | 9 (8.0) | 1.42 (0.33-6.15) |
| 70-91 | 119 | 95 (79.8) | 24 (20.2) | 0.56 (0.14-2.21) |
| **Highest education level**  **(years)** |  |  |  |  |
| ≤ 10 | 51 | 38 (74.5) | 13 (25.5) | Ref |
| 11-14 | 53 |  | <3 | 10.31 (2.04-52.14) |
| 15-16 | 56 | 48 (85.7) | 8 (14.3) | 1.67 (0.56-5.01) |
| >16 | 19 | 16 (84.2) | 3 (15.8) | 1.86 (0.41-8.36) |
| Vocational | 79 | 69 (87.3) | 10 (12.7) | 2.24 (0.79-6.34) |
| **Living alone (yes)** | 69 | 56 (81.2) | 13 (18.8) | 0.69 (0.29-1.66) |
| No | 189 | 166 (87.8) | 23 (12.2) | Ref |
| **Having children (yes)** | 74 | 58 (78.4) | 16 (21.6) | 0.41 (0.18-0.91) |
| No | 184 | 164 (89.1) | 20 (10.9) | Ref |
| **Comorbidity: At least one**  **chronic condition** | 91 | 77 (84.6) | 14 (15.4) | 1.34 (0.59-3.01) |
| Not | 167 | 145 (86.8) | 22 (13.2) | Ref |
| **Cancer location** |  |  |  |  |
| Colon | 180 | 151 (83.9) | 29 (16.1) | Ref |
| Rectum | 78 | 71 (91.0) | 7 (9.0) | 2.07 (0.81-5.32) |
| **Cancer free at follow-up** | 239 | 204 (85.4) | 35 (14.6) | 0.20 (0.02-1.80) |
| No | 19 |  | <3 | Ref |
| Selected additional  tabulations by  randomization group |  |  |  |  |
| **Highest education level**  **(years)** |  |  |  |  |
| **Overall ≤ 10** | **51** | **38 (74.5)** | **13 (25.5)** |  |
| Usual care | 30 | 23 | 7 |  |
| Intervention | 21 | 15 | 6 |  |
| **Having children (yes)** | **74** | **58 (78.4)** | **16 (21.6)** |  |
| Usual care | 39 | 32 | 7 |  |
| Intervention | 35 | 26 | 9 |  |
| **Having children (no)** | **184** | **164 (89.1)** | **20 (10.9)** |  |
| Usual care | 90 | 83 | 7 |  |
| Intervention | 94 | 81 | 13 |  |
